# Context Availability and Sentence Availability Ratings for 3,000 English Words and their Association with Lexical Processing

**DOI:** 10.5334/joc.211

**Published:** 2022-03-09

**Authors:** Ellen Taylor, Kate Nation, Yaling Hsiao

**Affiliations:** 1 Oxford University, United Kingdom of Great Britain and Northern Ireland, GB

**Keywords:** Semantics, Visual word processing, Language production

## Abstract

Words that can be easily placed in contexts are more easily processed, yet norms for context availability are limited. Here, participants rated 3,000 words for context availability and sentence availability, a new metric predicted to capture information relating to textual variation. Both variables were investigated alongside other word-level characteristics to explore lexical-semantic space. Analyses demonstrated that context availability and sentence availability are distinct. Context availability covaries with concreteness and imageability, while sentence availability captures information relating to contextual variation, frequency and ambiguity. Analyses of megastudy data showed that both context availability and sentence availability uniquely facilitated lexical decision performance.

Some words are easier to contextualise than others. This is captured by context availability, a metric that refers to how easily people can think of an imagined situation or circumstance for a word (Altarriba et al., 1999). Words high in context availability (CA) are advantaged in lexical decision and word naming (Colombo & Burani, 2002; Schwanenflugel & Noyes, 1996), and these effects are not readily explained by other psycholinguistic variables (Schwanenflugel et al., 1988). CA is closely related to concreteness and imageability (Van Hell & De Groot, 1998; Schwanenflugel et al., 1992). Variation in these variables is associated with variation in semantic richness (Yap et al., 2015).

As well as variation in imagined contexts, words vary in terms of the linguistic context in which they occur. This variation is captured by corpus-derived measures of contextual and semantic diversity (for reviews, see Caldwell-Harris, 2021; Jones et al., 2017). Adelman et al. (2006) used the term contextual diversity to describe document count (the number of unique texts a word appears in across a large corpus) and found this to be a better predictor of lexical decision and word naming than frequency. Other researchers have derived measures of semantic diversity that capture semantic variability in the contexts in which a particular word is used, not just the number of unique texts (Hoffman et al., 2013; Johns et al., 2012). While semantic diversity is also associated with lexical processing across a range of tasks (e.g., Hoffman & Woollams, 2015; Hsiao et al., 2020), there is some debate about its nature. For example, Cevoli et al. (2021) argued that semantic diversity as measured by Hoffman et al. (2013) is a general index of textual variation (i.e., capturing information about the text in which a word occurs rather than distinct meanings of words), whereas Hoffman et al. (2021) showed that semantic diversity also provides useful information about contextual variability in a word’s meaning. Recent work by Johns and Jones (2022) also demonstrates the need to consider the semantic content of contextual experience (and see Johns (2021) and Johns et al. (2021) for further discussion). Taken together, there is strong evidence that contextual experience shapes lexical organisation beyond both raw frequency and document count.

Our starting point in this paper is with the relationship between context availability (CA) and semantic diversity. Both measures index contextual experience, but in rather different ways, with CA being a subjective measure of how easily a situational context can be activated, and semantic diversity being derived from large corpora based on linguistic context. The relationship between these variables is unclear in the literature. In their analysis of 200 concrete and 200 abstract words, Moffat et al. (2015) reported a positive correlation between rated CA and semantic diversity. In contrast, Hoffman et al. (2013) reported a negative correlation between the two variables, based on 279 words. These contradictory findings might be due to limitations in the number and range of words sampled. To date, the largest set of openly accessible CA norms in English contains only 325 words (Altarriba et al., 1999). Much larger item-sets are now commonplace for other variables, allowing researchers to explore relationships with other lexical statistics and with behavioural data from megastudies (e.g., Keuleers & Balota, 2015). Our first aim was to produce CA ratings for a larger set of words, namely the 3,000 English words in Cortese and Fugett’s (2004) imageability norms, to facilitate research on CA and its relationship with other lexical variables.

Further exploration of larger data sets is warranted, but it seems likely that the linguistic context captured by a word’s semantic diversity is not the same as its rated context availability. Words may occur across diverse contexts in large language corpora for different reasons: as noted by Hoffman et al. (2020), high semantic diversity words are associated with a range of semantic states by virtue of their contextual promiscuity. Some words may be high in semantic diversity because they are polysemous (e.g., *glass*) and therefore have flexible meanings depending on the context. Other instances of high semantic diversity include function words that also depend on context for precise meaning. Given this semantic flexibility and openness, it is not surprising that there is a negative correlation between semantic diversity and measures of semantic richness such as imageability and concreteness (according to Hoffman et al. 2013, r = –.48 and r = –.51, respectively). In contrast, CA is positively associated with concreteness and imageability (Moffat et al., 2015) as words high in these variables can more easily activate situational contexts.

As well as indexing different aspects of context, it is important to note that semantic diversity and CA are very different types of measure. Semantic diversity, while objective, is derived from large corpora that do not reflect the language experience of any individual. By contrast, CA is obtained from participant ratings and is necessarily subjective. With this observation as a backdrop, our second aim was to investigate a new variable, sentence availability (SA). In contrast to CA in which participants rate how readily a context or circumstance comes to mind, SA invites people to indicate how easy it is to think of a sentence for each word. Our aim was to develop a measure of linguistic availability based on individual subjective reporting. This would allow us to directly compare situational and linguistic availability for the same words, and to consider how both relate to other lexical variables, and to lexical processing itself.

We included a range of lexical variables to help understand how CA and SA operate in lexical-semantic space. Core variables included frequency (Van Heuven et al., 2014) and document count (Hoffman et al., 2013), both of which correlated positively with CA and semantic diversity in previous studies. Words learned early in life tend to have high CA (Hoffman et al., 2013; Moffat et al., 2015) and they also tend to be higher in semantic diversity. We therefore included age of acquisition (Kuperman et al., 2012). Including imageability (Cortese & Fugett, 2003) and concreteness (Brysbaert et al., 2014) allowed us to test relationship between both types of availability and semantic richness. For completeness, we also considered variables that tap emotion and embodiment, including measures of valence (a word’s pleasantness), arousal (the intensity of emotion associated with a word), dominance (the extent to which a reader feels influential or in control in response to reading a word, ranging from *controlled* to *in control*) from Warriner et al. (2013), and body-object interaction (BOI, the ease with which a word can be physically interacted with) from Tillotson et al. (2008).

***Figure 1*** summarises the predicted relationships between our core variables of interest. The relationship between CA and concreteness is well established (Van Hell & De Groot, 1998; Schwanenflugel et al., 1992). We therefore expected CA to correlate with concreteness and imageability. Sentence availability might not be so closely aligned with concreteness and imageability because it draws upon linguistic contexts. We therefore predicted that SA would be related to variables capturing linguistic variation, such as semantic diversity. To pre-empt our findings with an example, ‘braid’ had high CA relative to SA, suggesting that it can be associated with a context more easily than a sentence. In contrast, ‘fleck’ was more easily placed in a sentence than a context (see ***Figure 2*** for further examples). ‘Braid’ is imageable and concrete, but only appears in a limited number of contexts. ‘Fleck’, however, is more difficult to associate with a particular context, but it can be placed in a sentence with relative ease. It is less imageable than ‘braid’ but is more diverse as it appears in a wider range of linguistic contexts.

**Figure 1:**
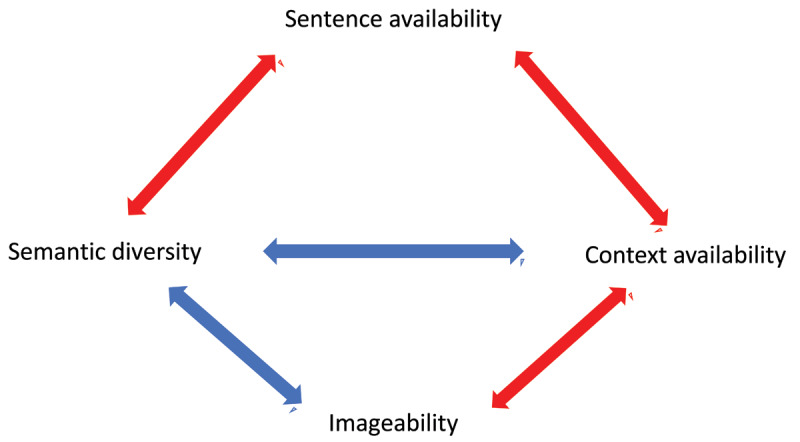
Predicted relationships between context availability, sentence availability, imageability and semantic diversity. Negative relationships are shown in blue, and positive relationships in red.

**Figure 2:**
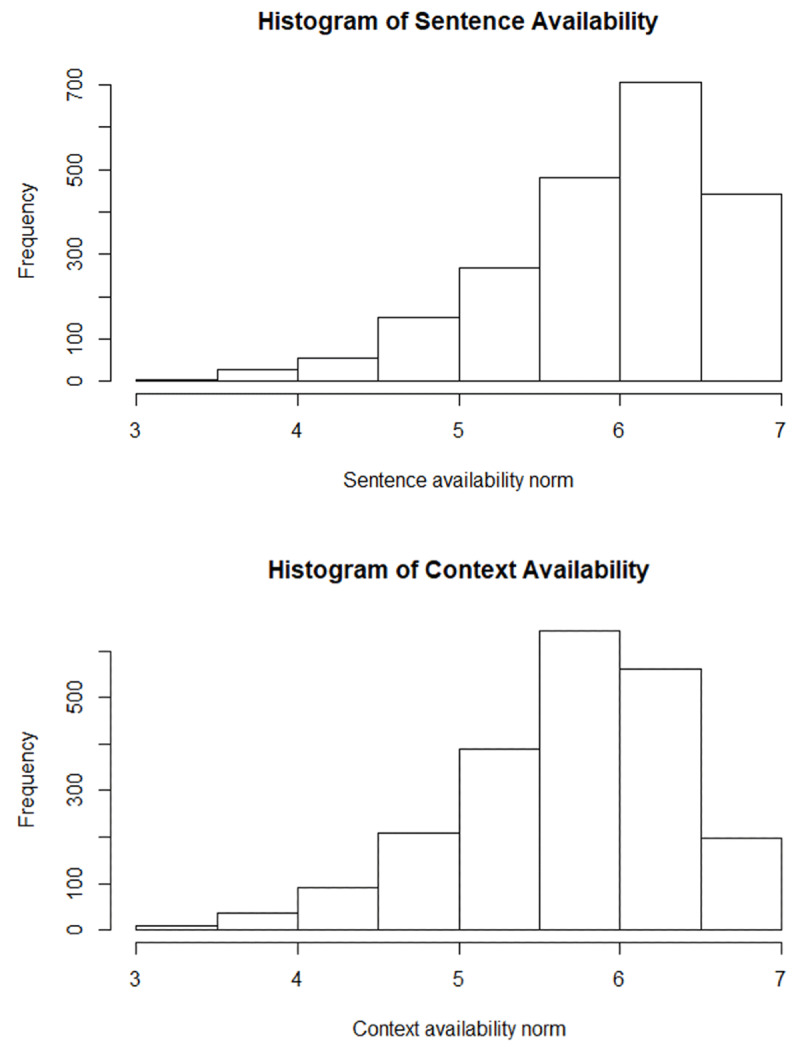
Histograms showing the distribution of CA (M = 5.69, SD = 0.68) and SA (M = 5.94, SD = 0.67).

Based on findings reported by Hoffman et al. and Moffat et al., we expected CA to correlate positively with word frequency and document count, and negatively with age of acquisition. As discussed by Pexman (2020), semantic concepts are learnt largely through sensorimotor association and, in the absence of these associations, meaning can be grounded in felt experience. In line with this, Moffat et al. (2015) reported positive correlations between CA and body-object interaction and emotional experience. Given SA might be more associated with linguistic diversity, we anticipated that it would be less associated with emotion and embodiment variables than CA.

Finally, we tested whether CA and SA are associated with lexical processing, using lexical decision data from the British Lexicon Project (BLP; Keuleers et al., 2012) and the English Lexicon Project (ELP; Balota et al., 2007). We hypothesised that both types of availability would facilitate lexical decision, and both would explain unique variance in performance.

## Method

### Participants

Eight hundred native speakers of British English were recruited via Prolific (Palan & Schitter, 2018) to provide either context availability or sentence availability ratings. After exclusions (see below) we analysed data from 359 people rating contexts and 365 rating sentences.

### Materials and Procedure

Ratings were collected for the 3,000 English monosyllabic words in Cortese and Fugett’s (2004) imageability norms, programmed using the online platform Gorilla (*www.gorilla.sc*). We created 20 150-word lists, sampled from the 3,000 itemset. Each list contained 6 non-words, randomly placed to check for attention. Participants in each version (CA or SA) were randomly assigned to one of the 20 lists. The words were presented in random order in six blocks of 26 words. They were asked to rate each word on a 7-point Likert scale. The lower end of this scale was labelled ‘easy to think of a context’ (CA) or ‘easy to think of a sentence’ (SA). The other end of the scale was ‘difficult to think of a context/sentence’. Participants were told to skip words they did not know. Due to randomisation via Prolific, some word-lists were allocated additional participants. This resulted in each word being rated by a maximum of 21 people for context availability and 20 people for sentence availability.

## Results

Participants who provided ratings for more than 30% of the catch non-words or gave the same rating for 95% of trials were removed, N = 41 (CA) and 35 (SA). Words with less than 15 valid ratings for both variables (N = 509, 16.97% of data) were then excluded from the analyses reported here (but data for all 3,000 words are available at *https://osf.io/gr73b/*). The mean (SD) number of observations per word was 17.73 (1.70) for CA and 17.81 (1.48) for SA. Ratings were reverse coded to follow the same direction as Altarriba et al.’s norms, with higher scores reflecting higher availability. Both CA and SA were normally distributed (***Figure 2***) but skewed towards the upper end of the scale. They correlated with each other, r = .643, p < .001, and CA correlated with CA for the same items in Altarriba et al.’s dataset, N = 117, r = .645, p < .001 (for further information see *https://osf.io/gr73b/*).

**Figure 3:**
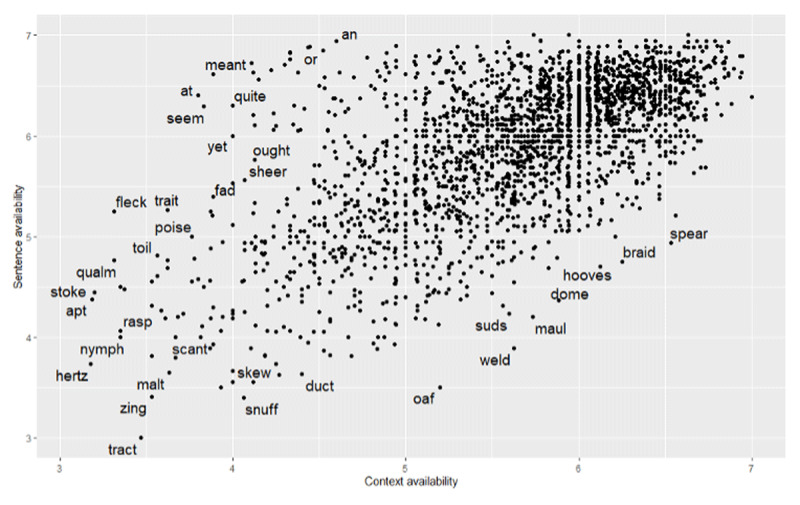
Scatterplot showing the relationship between context availability and sentence availability, with examples labelled.

**Figure 4:**
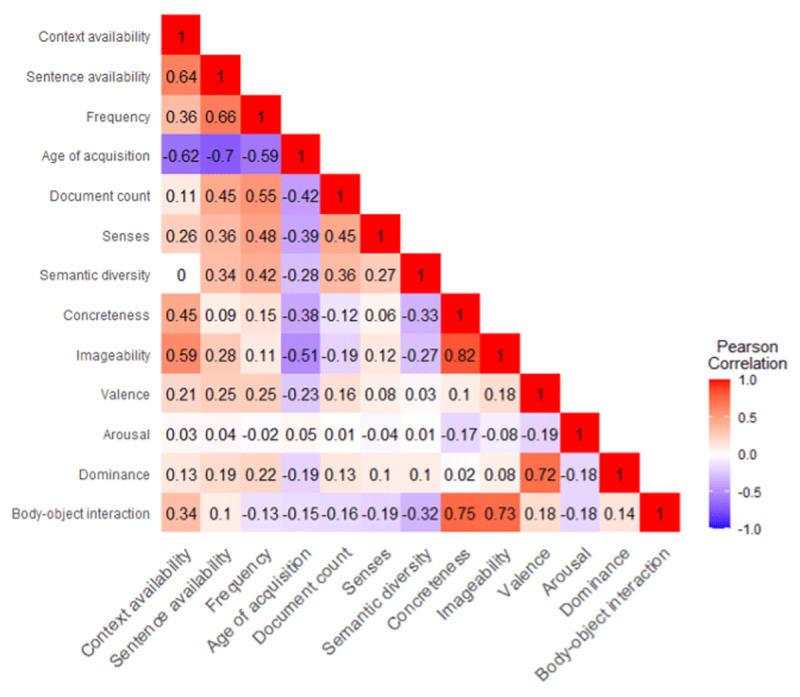
Pearson correlations for CA, SA and other lexical variables, based on N available for each pairwise correlation.

Availability norms are plotted against each other in ***Figure 3***, with examples labelled to illustrate the relationship between CA and SA. ***Figure 4*** shows Pearson correlations between CA, SA, and a range of other variables. Of note, there was a positive correlation between CA and imageability, r = .59, p < .001 but not between CA and semantic diversity, r = <–.001, p = .945. In contrast, SA was positively correlated with both imageability, r = .28, p < .001 and semantic diversity, r = .34, p < .001. Both availability measures correlated positively with frequency (r = .36 for CA; r = .66 for SA) and negatively with age of acquisition (r = –.62 for CA; r = –.70 for SA).

Having observed no correlation between CA and semantic diversity, we checked the relationship for those items also included by Hoffman et al. (2013). They reported a negative correlation (N= 325; r = –.26), an observation that replicated for the N=117 Hoffman words also included here, r = –.325. This observation indicates that the itemset used in Hoffman et al.’s analyses might be too small to accurately reflect the relationship between CA and semantic diversity.

**Figure 5:**
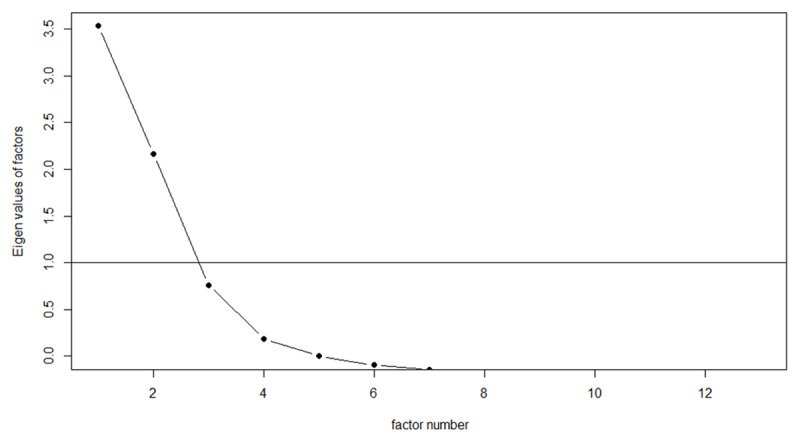
Scree plot showing the factors identified by parallel analysis. Note that only Factors 1 and 2 had Eigen values above 1.0.

To consider the relationships between the variables further, we used factor analysis with oblique rotation, using the function ‘oblimin’ in R Package ‘Psych’ (Revelle, 2017). Similar results were seen following varimax rotation. Parallel analysis identified three factors (***Table 1***; see ***Figure 5*** for scree plot) that we labelled as ‘occurrence’ which captured variables relating to frequency and ambiguity; ‘richness’ which captured imageability and concreteness information; and ‘affect’, with positive loadings from valence and dominance. Occurrence explained 24.3% of the variance, richness 22.5%, and affect 11.5%. There were small but significant correlations between the factors. CA loaded positively onto both occurrence and richness, while SA loaded only on occurrence.

**Table 1:** Factor loadings for an exploratory factor analysis using oblique rotation. Only words with data for all variables are included (N = 1,270). Loadings less than 0.3 are not displayed; lower part of table shows correlation between factors.


	OCCURRENCE	RICHNESS	AFFECT

CA	0.501	0.487	

SA	0.801		

Zipf Freq	0.801		

AoA	–0.774		

Doc Count	0.626		

Senses	0.574		

SemD	0.497	–0.424	

Concreteness		0.874	

Imageability		0.936	

Valence			0.824

Arousal			

Dominance			0.857

BOI		0.808	

	**OCCURRENCE**	**RICHNESS**	**AFFECT**

Occurrence		.15	.24

Richness	.15		.12

Affect	.24	.12	


We then investigated whether CA and SA predicted lexical decision, using linear mixed effects models (lme4 in R, Bates et al., 2007; p values from lmerTest, Kuznetsova et al., 2017) and data from the BLP (Keuleers et al., 2012). Accuracy was analysed using glmer and the RT analysis used lmer. Both models included frequency, age of acquisition, semantic diversity and imageability (variables that influence lexical decision) as well as CA and SA. Random intercepts of word and participant were included, and all predictor variables were centered and scaled. As shown in ***Table 2***, both availability measures were associated with lexical decision performance. Comparisons demonstrated that this model explained significantly more variance than when only one of the availability measures was included (for RT: x^2^(1) = 43.301, p < .001 compared to CA alone; x^2^(1) = 58.062, p < .001 compared to SA alone. For accuracy: x^2^(1) = 33.845, p < .001 compared to CA alone; x^2^(1) = 21.524, p < .001 compared to SA alone).

**Table 2:** Fixed effects predicting lexical decision accuracy (upper section) and RT (lower section in BLP data (N = 1724 words).


	ESTIMATE	SE	Z	P	

Intercept	2.99045	0.14370	20.810	<.001	

Freq	0.45056	0.06463	6.971	<.001	

AoA	0.06837	0.06679	1.024	.306	

SemD	0.15179	0.04844	3.133	.002	

Imageability	0.27313	0.05048	5.410	<.001	

CA	0.25046	0.05375	4.660	<.001	

SA	0.33520	0.05763	5.817	<.001	

	**ESTIMATE**	**SE**	**DF**	**T**	**P**

Intercept	586.037	11.259	37.502	52.052	<.001

Freq	–18.910	1.628	2054.430	–11.615	<.001

AoA	5.007	1.723	2042.398	2.905	.004

SemD	–3.538	1.325	2094.082	–2.671	.008

Imageability	–4.962	1.256	2039.830	–3.951	<.001

CA	–10.991	1.435	2068.772	–7.660	<.001

SA	–10.501	1.590	2092.945	–6.602	<.001


*Note*: Model structure: LD ~ Freq + AoA + SemD + imageability + context availability + sentence availability + (1|participant) + (1|word).

The generalisability of these results was tested using data from the ELP (Balota et al., 2007). Once again, both availability measures were associated with lexical decision performance (***Table 3***).

**Table 3:** Fixed effects predicting lexical decision accuracy (upper section) and RT (lower section in ELP data (N = 2119 words).


	ESTIMATE	SE	Z	P	

Intercept	–2.44269	0.45169	–5.408	<.001	

Freq	0.42106	0.04631	9.092	<.001	

AoA	–0.05054	0.01619	–3.122	.002	

SemD	0.68378	0.10889	6.279	<.001	

Imageability	0.28597	0.02624	10.896	<.001	

CA	0.14105	0.05627	2.507	.012	

SA	0.22429	0.05900	3.802	<.001	

	**ESTIMATE**	**SE**	**DF**	**T**	**P**

Intercept	977.2004	25.2271	2236.1832	38.736	<.001

Freq	–28.7873	2.3931	2103.7453	–12.029	<.001

AoA	3.2613	0.8641	2099.2646	3.774	<.001

SemD	–29.0695	5.9157	2098.5337	–4.914	<.001

Imageability	–12.0402	1.3371	2098.8602	–9.005	<.001

CA	–7.3309	2.9978	2099.0068	–2.445	.015

SA	–14.1835	3.2280	2100.8502	–4.394	<.001


*Note*: Model structure: LD ~ Freq + AoA + SemD + imageability + context availability + sentence availability + (1|participant) + (1|word).

## Discussion

Context and sentence availability values were collected for 3,000 monosyllabic words. While correlating positively (r = .643), changing the instruction to focus on sentences rather than contexts does change the nature of availability. The two variables showed a different pattern of correlations with other variables. CA was positively correlated with imageability but not correlated with semantic diversity whereas SA was positively correlated with both diversity and imageability. SA showed strong relationships with a range of variables (including frequency, age of acquisition, and document count), while CA was closely related to concreteness and imageability. Three factors were identified by factor analysis, reflecting ‘occurrence’, ‘richness’, and ‘affect’. As anticipated, CA loaded onto richness, but also onto occurrence. Semantic diversity also loaded in the same direction as CA for occurrence, but the opposite direction for richness. This highlights a more complex relationship between CA and semantic diversity than apparent from the pattern of simple correlations. The observation that SA loaded onto occurrence supports the hypothesis that it might capture information relating to textual variation and thus reflects word usage in sentences. This is also consistent with its correlation with semantic diversity. In contrast, ratings of concreteness and imageability reflect the semantic richness of words, and this might be less directly related to linguistic diversity, where more abstract or underspecified words tend to occur across varying contexts. While we collected a large set of availability ratings, we did not ask participants to produce the imagined sentences they associated with target words. A sentence production task would allow for a more thorough investigation of this.

Both availability measures were negatively correlated with age of acquisition, with words learnt earlier in life being easier to contextualise (see Hills et al. (2010) for broader discussion of contextual diversity and early word learning). It is possible that other variables closely related to age of acquisition might be influencing how participants rate words for CA and SA. For instance, word familiarity is also negatively correlated with age of acquisition (Stadthagen-Gonzalez & Davis, 2006). Greater familiarity might make words easier to associate with sentences and contexts.

Both CA and SA predicted lexical decision performance. This finding held for both BLP and ELP datasets, demonstrating that high availability is associated with more efficient lexical processing. The contribution of SA could be driven by shared variance with other lexical variables (e.g., number of senses, valence, dominance, and arousal) which were not included in our model, or by the high SA ratings for function words such as ‘or’ (6.85), ‘an’ (6.94) and ‘at’ (6.40; see ***Figure 3***). SA tends to be high both for function words which are contextually flexible, and for words which are semantically rich. CA is also associated with semantic richness, but tends to be lower for function words as these are harder to place in a situation context than content words. In a post-hoc analysis, we identified 83 function words in our dataset. All analyses patterned identically after excluding these words (for details, see *https://osf.io/gr73b/*). This suggests that function words are not skewing the pattern of results seen here. The finding that the two availability measures account for separate variance in lexical processing supports the conclusion drawn from the correlational analysis and factor analysis, namely that SA and CA reflect different types of availability information.

While SA was associated with semantic diversity, there are clear differences between these measures: SA was correlated with CA and imageability, while semantic diversity was uncorrelated with CA and negatively correlated with imageability. In other words, words that easily arouse imagery may be easy to place within sentences, but they might not be semantically flexible across contexts. Although SA did not load onto ‘richness’ alongside imageability, it is nonetheless positively correlated with this variable, suggesting that SA captures information other than linguistic variation. As SA is based on participant ratings, it might be more influenced by the perceived semantic richness of the to-be-rated words, relative to semantic diversity which is corpus derives and reflects word co-occurrence.

Before closing, we should note some limitations of our study. For both measures, participants gave high ratings suggesting that most words were easily associated with a context or sentence. While the norms collected by Altarriba et al. (1999) were also rated highly, this was more pronounced in our study. This might reflect participant differences, or differences across the item-sets; following Cortese and Fugett, our words were all monosyllabic, but as those authors noted, many studies tend to focus on monosyllabic words, making this a useful item-set for future experiments. That said, there is a clear need to extend the number and range of words considered, not least because multisyllabic words are more complex and may relate to availability within semantic space in different ways. We note too that we excluded words that received less than 15 ratings. There is little consensus in previous studies as to the number of participants needed to produce valid ratings. In Kuperman et al.’s (2012) age of acquisition norms, each word had 18 or more ratings, drawn from a large participant pool. A different approach is for a number of people to rate all words (e.g., 31 participants for Cortese & Fugett, 2004; 78 for Altarriba et al., 1999). In our study, participants had the option to skip words that they did not know, adding reassurance that the ratings were a valid reflection of availability.

In conclusion, CA and SA are two distinct measures that capture word knowledge and word usage in different ways. CA captures information similar to concreteness and imageability, while SA is more closely related to textual or linguistic variation. Words high in either type of availability showed processing advantages in lexical decision, and each explained unique variance when other key lexical variables were controlled.
